# The fate and lifespan of human monocyte subsets in steady state and systemic inflammation

**DOI:** 10.1084/jem.20170355

**Published:** 2017-07-03

**Authors:** Amit A. Patel, Yan Zhang, James N. Fullerton, Lies Boelen, Anthony Rongvaux, Alexander A. Maini, Venetia Bigley, Richard A. Flavell, Derek W. Gilroy, Becca Asquith, Derek Macallan, Simon Yona

**Affiliations:** 1 Division of Medicine, University College London, University of London, London, England, UK; 2 Institute for Infection and Immunity, St. George’s, University of London, London, England, UK; 3 Theoretical Immunology Group, Faculty of Medicine, Imperial College London, London, England, UK; 4 Department of Immunobiology, Yale University, New Haven, CT; 5 Howard Hughes Medical Institute, Yale University, New Haven, CT; 6 Newcastle University Medical School, Newcastle University, Newcastle Upon Tyne, England, UK; 7 St. George’s University Hospitals NHS Foundation Trust, London, England, UK

## Abstract

In humans, the monocyte pool comprises three subsets (classical, intermediate, and nonclassical) that circulate in dynamic equilibrium. The kinetics underlying their generation, differentiation, and disappearance are critical to understanding both steady-state homeostasis and inflammatory responses. Here, using human in vivo deuterium labeling, we demonstrate that classical monocytes emerge first from marrow, after a postmitotic interval of 1.6 d, and circulate for a day. Subsequent labeling of intermediate and nonclassical monocytes is consistent with a model of sequential transition. Intermediate and nonclassical monocytes have longer circulating lifespans (∼4 and ∼7 d, respectively). In a human experimental endotoxemia model, a transient but profound monocytopenia was observed; restoration of circulating monocytes was achieved by the early release of classical monocytes from bone marrow. The sequence of repopulation recapitulated the order of maturation in healthy homeostasis. This developmental relationship between monocyte subsets was verified by fate mapping grafted human classical monocytes into humanized mice, which were able to differentiate sequentially into intermediate and nonclassical cells.

## Abstract (precis)

Using stable isotope labeling, Patel et al. establish the lifespan of all three human monocyte subsets that circulate in dynamic equilibrium; in steady state, classical monocytes are short-lived precursors with the potential to become intermediate and nonclassical monocytes. They highlight that systemic inflammation induces an emergency release of classical monocytes into the circulation.

## Introduction

The mononuclear phagocyte system comprises three types of cells: monocytes, macrophages, and DCs, as well as their committed bone marrow progenitors (van Furth et al., 1972; Yona and Gordon, 2015). Collectively, the cells of the mononuclear phagocyte system play key functions in maintaining tissue homeostasis during steady state as well as orchestrating the genesis and resolution of the immune response (Davies et al., 2013; Wynn et al., 2013; Ginhoux and Jung, 2014).

It is now recognized that the majority of tissue macrophage populations are seeded before birth (Ginhoux et al., 2010; Schulz et al., 2012; Guilliams et al., 2013; Hashimoto et al., 2013; Yona et al., 2013; Mass et al., 2016) and maintained via self-proliferation throughout adulthood with minimal monocyte input (Soucie et al., 2016). Conversely, DCs and monocytes arise from distinct adult hematopoietic stem cell precursors in the bone marrow (Fogg et al., 2006; Naik et al., 2007; Onai et al., 2007, 2013; Liu et al., 2009; Hettinger et al., 2013; Breton et al., 2015; Lee et al., 2015).

Circulating monocytes represent a versatile and dynamic cell population, composed of multiple subsets which differ in phenotype, size, morphology, and transcriptional profiles and are defined by their location in the blood (Geissmann et al., 2003; Cros et al., 2010; Ingersoll et al., 2010; Wong et al., 2011; Mildner et al., 2013a). These discrete monocyte subsets can be distinguished by the expression of CD14 and CD16 in humans and Ly6C, CCR2, and CX_3_CR1 in mice (Ziegler-Heitbrock et al., 2010). In humans, CD14^+^ CD16^−^ (classical) monocytes make up ∼85% of the circulating monocyte pool, whereas the remaining ∼15% consist of CD14^+^ CD16^+^ (intermediate) and CD14^lo^ CD16^+^ (nonclassical) monocytes (Passlick et al., 1989; Wong et al., 2011). Similarly, in mice, two populations of monocytes have been described: Ly6C^hi^ CCR2^+^ CX_3_CR1^int^ and Ly6C^lo^ CCR2^−^ CX_3_CR1^hi^, representing classical and nonclassical monocytes, respectively (Geissmann et al., 2003). Monocyte egression from the bone marrow requires expression of the chemokine receptor CCR2, which is restricted to classical monocytes (Shi and Pamer, 2011).

Classical monocytes are rapidly recruited to sites of infection (Serbina and Pamer, 2006; Liao et al., 2017) and injury (Nahrendorf et al., 2007; Zigmond et al., 2014), where they exhibit considerable functional plasticity (Arnold et al., 2007; Avraham-Davidi et al., 2013). Interestingly, classical monocytes replenish resident peripheral monocyte–derived cells under steady-state conditions (Varol et al., 2009; Tamoutounour et al., 2013; Bain et al., 2014; Guilliams et al., 2014). Nonclassical monocytes have been proposed to act as custodians of vasculature by patrolling endothelial cell integrity in an LFA-1–dependent fashion (Auffray et al., 2007).

During steady state, rodent blood monocyte subsets represent stages of a developmental sequence; classical monocytes have been shown to convert into nonclassical monocytes over time (Sunderkötter et al., 2004; Yrlid et al., 2006; Varol et al., 2007; Yona et al., 2013; Thomas et al., 2016). However, it remains to be shown what, if any, relationships exist among the three principal human monocyte subsets and how long each of these subsets resides in the circulation.

Although the vast majority of information concerning mononuclear phagocyte ontogeny, function, and kinetics is derived from mouse studies, due to the challenging nature of performing studies in a clinical setting, some important insights into human monocyte biology have been gained from studying pathological states. Patients with a GATA2 mutation (encoding the GATA-binding protein 2) have an absence of all blood monocytes; despite this, their resident dermal and lung macrophages remain unaffected, suggesting that the development of these populations is independent of blood monocytes (Bigley et al., 2011). Interestingly, patients with rheumatoid arthritis exhibit an increase in circulating intermediate monocytes (Cooper et al., 2012). Furthermore, stroke patients have been reported to increase their intermediate monocytes 2 d after their initial insult, and this increase inversely correlated with mortality (Urra et al., 2009). These data raise the questions of whether and how circulating human monocyte subsets are related, how long each population circulates, and what impact inflammation has on this process.

Fifty years ago, van Furth and Cohn performed a series of elegant studies examining monocyte dynamics in rodents with ^3^H-thymidine. They concluded that monocytes transit from the bone marrow to the blood, with a circulating half-life of ∼22 h (van Furth and Cohn, 1968). More recently, studies in mice demonstrated that classical monocytes have a half-life of <1 d before converting into nonclassical monocytes with a half-life of ∼2.2 d (Yona et al., 2013). Nevertheless, the fate and kinetics of human monocyte subsets under steady state and inflammation remain to be resolved. A major breakthrough in examining in vivo human leukocyte kinetics came with the advent of nontoxic stable isotope labeling approaches (Macallan et al., 1998; Busch et al., 2007). Specifically, the deuterium from deuterium-labeled glucose or heavy water incorporates stably into the backbone of DNA of dividing cells. The deuterium-glucose labeling approach is particularly suited to the study of rapidly dividing cells and has been applied in humans to study the turnover of T cell populations such as regulatory T cells (Vukmanovic-Stejic et al., 2006), to memory T cell subsets in HIV infection (Zhang et al., 2013), and, more recently, to cells of the innate immune system, such as neutrophils (Lahoz-Beneytez et al., 2016).

Here, we report a series of studies investigating the development and kinetics of human monocyte subpopulations. We hypothesized that the fate and kinetics of the three monocyte subsets (classical, intermediate, and nonclassical) were intimately linked and could be defined in kinetic terms. We first investigated the steady-state kinetics in healthy human volunteers using in vivo deuterium-labeled glucose as a precursor. We then repeated these studies in the context of endotoxin-induced systemic inflammation, where we observed a transient depletion of almost the entire circulating monocyte pool; in this way, we were able to study the early repopulation of an “empty” blood compartment. Finally, we tested our sequential development hypothesis in the humanized MISTRG mouse (Rongvaux et al., 2014) to build a comprehensive picture of how human monocyte subsets are regulated in steady state and systemic inflammation.

## Results and discussion

### Characterization of human monocyte subset kinetics under steady state

**Figure 1.**
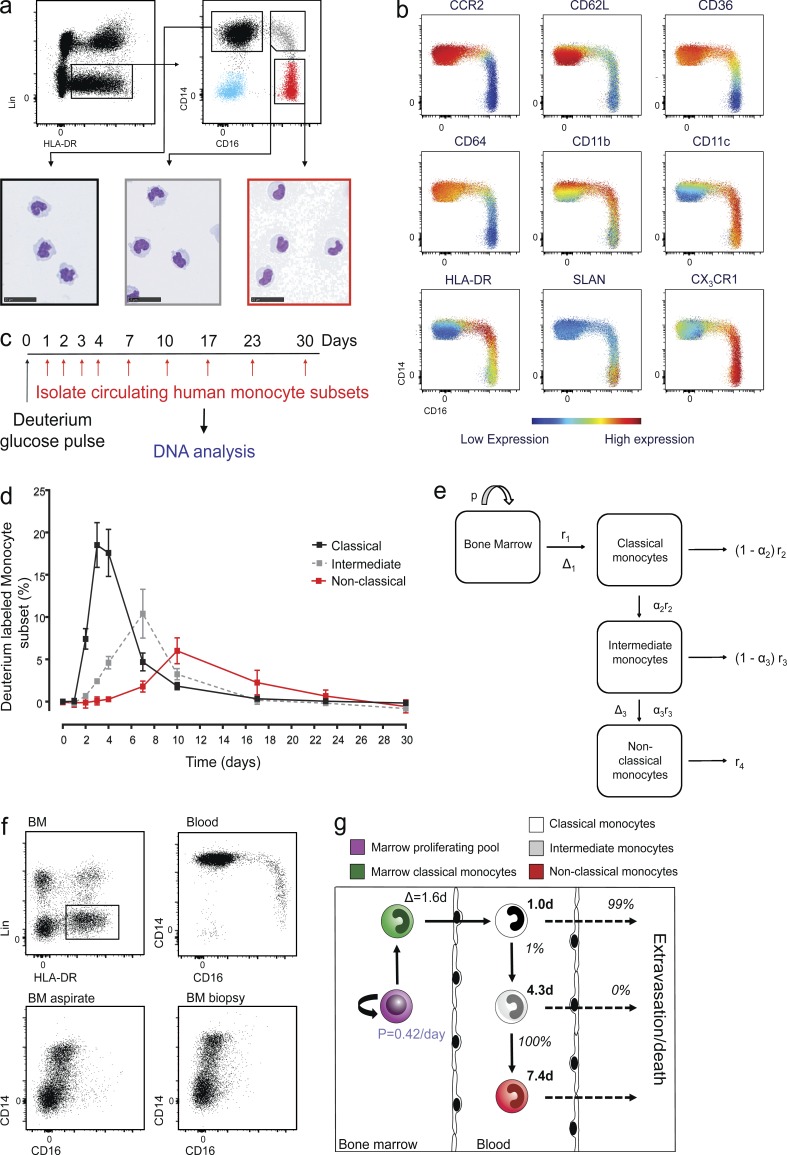
**In vivo labeling and a methodological approach of modeling human monocyte subset kinetics at steady state.** (a) Polychromatic flow cytometry gating strategy for blood monocyte subsets. Peripheral blood mononuclear phagocyte cells were identified as Lin^−^ (CD3, CD19, CD20, CD56, CD66b) HLA-DR^+^ cells. This population comprises classical monocytes (CD14^+^ CD16^−^: black gated population), intermediate monocytes (CD14^+^ CD16^+^: gray gated population), and nonclassical monocytes (CD14^lo^ CD16^+^: red gated population) representative of >10 subjects. Representative cytospin images from 10 healthy volunteers stained with hematoxylin and eosin (bottom). Bar, 25 µm. (b) Flow cytometry viSNE analysis of monocyte subsets illustrating membrane expression for CCR2, CD62L, CD36, CD64, CD11b, CD11c, HLA-DR, SLAN, and CX_3_CR1, representative of eight healthy volunteers. (c) Schematic of protocol for labeling newly divided cells. Healthy volunteers received 20 g deuterium-labeled glucose over 3 h. Monocytes subsets were then sorted from whole blood over a 30-d period, DNA was extracted to quantify the deuterium enrichment in each monocyte subset by gas chromatography mass spectrometry. (d) Percentage of deuterium label in peripheral blood classical (black), intermediate (gray), and nonclassical (red) monocytes following oral admission of deuterated glucose in four healthy volunteers; values shown are mean ± SEM. (e) Model of circulating monocyte kinetics. Cartoon depicts the sequential model for the fate of circulating monocyte subsets. Monocytes mature in the bone marrow, where their precursors proliferate at a rate p. Classical monocytes leave the bone marrow at rate r_1_, after a delay of Δ_1_ days between the last proliferation and release into the circulation. In the blood, classical monocytes either mature into intermediate monocytes at rate α_2_ r_2_, where α = proportion of the subset, or they disappear from the blood (either by death or by moving to other organs) at rate (1–α_2_)r_2_. The total disappearance rate is thus r_2_. Likewise, a proportion α_3_ of the intermediate monocyte subset develop into nonclassical monocytes, the remainder disappearing from blood. A parameter Δ_3_ has been included to allow for a potential delay in the differentiation of intermediate monocyte to nonclassical monocytes. (f) Polychromatic flow cytometry comparing BM with circulating monocyte subsets. Human BM was initially gated as Lin^−^HLA-DR^+^. Human BM obtained as either an aspirate or femoral head excavated biopsy was examined by flow cytometry to identify resident monocyte subsets. Only classical monocytes could be detected in the biopsy specimen. These data are representative of three donors for each procedure. (g) Summary of the steady-state kinetics for monocyte subsets. Figures in black bold text denote lifespans in each compartment figures in italics denote the relative probability of each cell undergoing the respective fate (death/disappearance versus phenotype transition). Progenitor cells in the bone marrow proliferate at rate of 0.42/d (blue), where the postmitotic cells remain within the bone marrow for 1.6 d before being released into the circulation as classical monocytes. Classical monocytes contribute 87% to the total monocyte pool, whereas intermediate and nonclassical monocytes make up 5% and 8%, respectively. 99% of classical monocytes leave the circulation, and 1% go onto become intermediate monocytes. 100% of intermediate monocytes mature in the circulation to become nonclassical monocytes under steady state.

The literature has not always clearly distinguished between monocyte subsets, making interpretation confusing. We chose to follow a systematic strategy to identify the three conventional monocyte subsets of interest. Lin^−^ (CD3, CD19, CD20, CD56, and CD66b) HLA-DR^+^ cells were separated into (1) CD14^+^CD16^−^ classical monocytes, (2) CD14^+^CD16^+^ intermediate monocytes, and (3) CD14^lo^CD16^+^ nonclassical monocytes (Fig. 1 a; Ziegler-Heitbrock et al., 2010). In addition to CD14 and CD16 expression, we confirmed additional membrane marker expression between monocyte subsets (Fig. 1 b and Fig. S1 a; Ingersoll et al., 2010). Interestingly, these data demonstrate the discrete nature of monocyte subsets is a continuum of more than just CD14 and CD16 expression.

To investigate monocyte kinetics under normal physiological homeostatic conditions, we administered a short pulse (3 h) of deuterium-labeled glucose (6,6-^2^H_2_-glucose) to healthy human volunteers and analyzed flow-sorted monocyte subsets at sequential time points thereafter for deuterium incorporation (Fig. 1 c; Macallan et al., 2009; Westera et al., 2013). Analysis of deuterium labeling data revealed that monocyte subsets exhibited a highly consistent pattern in all volunteers studied. Significantly, there was no deuterium labeling for the first 24 h after administration, consistent with a postmitotic “maturation” phase preceding release from bone marrow into the circulation. We then observed early integration of deuterium in classical monocytes, reaching a peak 3 d after labeling (Fig. 1 d). At these early time points, intermediate monocytes were also labeled with deuterium but at a much lower level than classical monocytes. No label was observed in nonclassical monocytes until day 7. This pattern of sequential appearance of labeling in human monocyte subsets is reminiscent of previous studies in experimental models in rodents, where classical monocytes convert into nonclassical monocytes over time (Sunderkötter et al., 2004; Varol et al., 2007; Yona et al., 2013; Gamrekelashvili et al., 2016).

**Figure 2.**
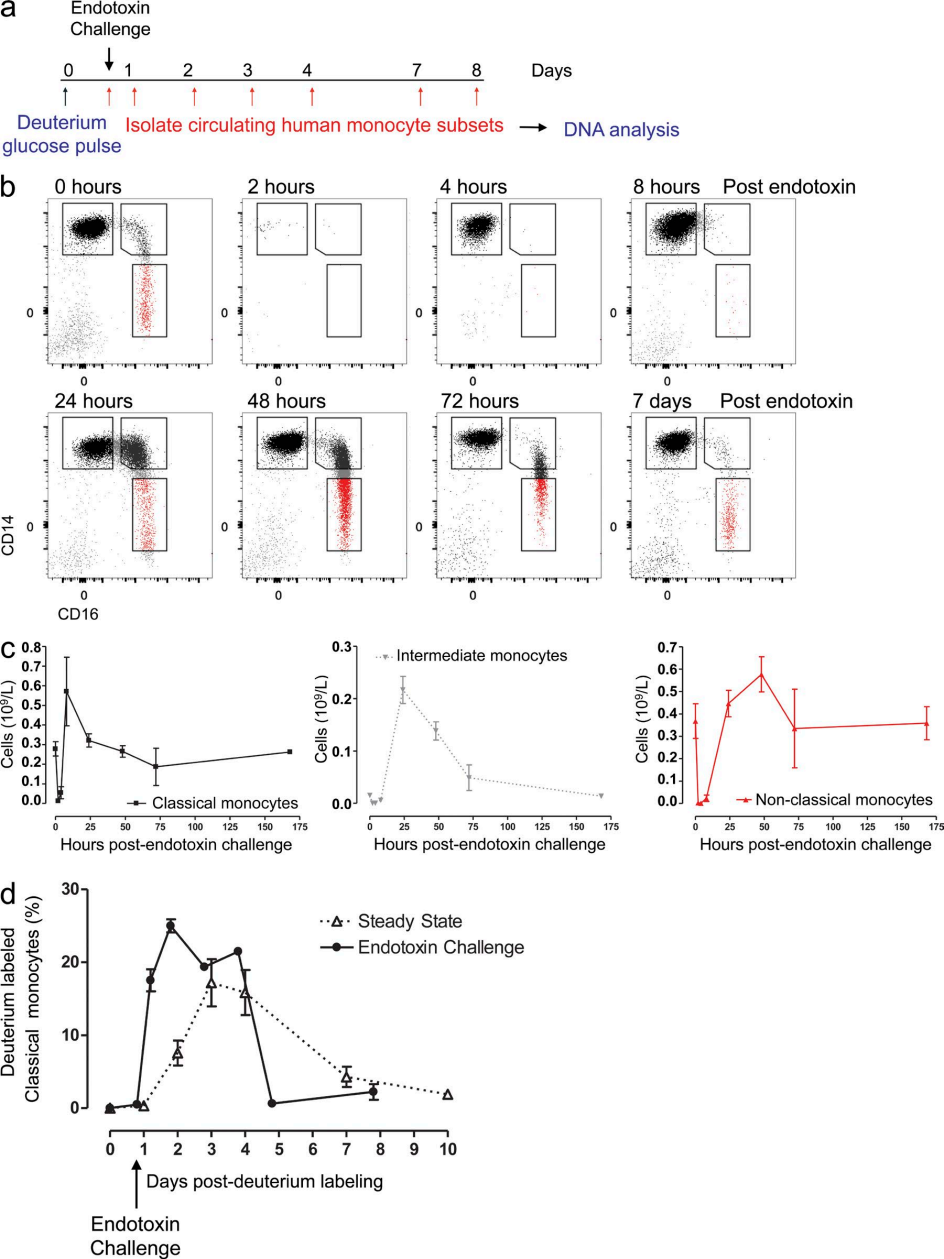
**Sequential reappearance of monocytes subsets after endotoxin challenge.** (a) Schematic protocol for administrating deuterium-labeled glucose 20 h before i.v. endotoxin 2 ng/kg in healthy volunteers. Classical monocytes were then sorted from whole blood, DNA extracted, and deuterium enrichment quantified by gas chromatography mass spectrometry over the ensuing 8 d. (b) Flow cytometry analysis of human monocyte subsets at 0, 2, 4, 8, 24, 48, and 72 h and 7 d after i.v. administration of endotoxin, representative of 10 individuals. (c) Time course of absolute monocyte numbers at selected time points following endotoxin challenge for classical, intermediate, and nonclassical monocytes (mean ± SEM × 10^9^/L of three individual subjects; note the different scale for each subset). (d) Comparison of deuterium-labeled classical monocyte egression from the BM under normal physiological conditions (triangles, dashed line, four subjects) and after endotoxin challenge (circles, solid line, three subjects). Values represent mean ± SEM.

This chronological acquisition of deuterium by circulating monocyte subsets is most likely to be explained by a sequential ontogeny scenario in which deuterium is incorporated into precursors that differentiate into classical monocytes in bone marrow; these classical monocytes are released into the circulation, where they undergo one of two fates: they either differentiate into intermediate monocytes or disappear by death or migration. Similarly, intermediate monocytes either leave the blood (by death or migration) or differentiate into nonclassical monocytes. The likelihood of each onward differentiation step (classical to intermediate and then intermediate to nonclassical) was denoted by the rate αr for each subpopulation, resulting in a corresponding rate of loss from the circulating pool (by death or migration) of (1-α)r. This model is summarized in Fig. 1 e. The alternative parallel ontogeny scenario was also considered; in this model, the three subsets arise from separate linages, each with its own distinct postmitotic kinetics. This model could certainly be made to fit the data mathematically, as it has so many free parameters, but was deemed unlikely on biological grounds. First, it predicts the presence of intermediate and nonclassical monocytes in the bone marrow, contrary to our observations where only classical monocytes were detected following bone marrow biopsy (Fig. 1 f; blood monocyte contamination could be detected in bone marrow aspirate), and second, because it would be inconsistent with information from studies in rodents (Sunderkötter et al., 2004; Yrlid et al., 2006; Varol et al., 2007; Yona et al., 2013; Gamrekelashvili et al., 2016). In the sequential model used here (Fig. 1 e) proliferation is restricted to the bone marrow; we excluded models in which circulating subsets proliferate in the blood on the basis of (1) the absence of any deuterium enrichment in such cells 24 h after labeling (Fig. 1 d) and (2) the absence of markers of cell cycling (Fig. S1 b).

Results from fitting the model to the experimental data are shown in Table 1 and Fig. S2. We found that classical monocytes have a very short circulating lifespan (mean 1.0 ± 0.26 d). Most cells leave the circulation or die, whereas the remaining cells transition to intermediate monocytes. Intermediate monocytes have a longer lifespan (mean 4.3 ± 0.36 d) and all transition to nonclassical monocytes. Nonclassical monocytes in turn have the longest lifespan in blood (mean 7.4 ± 0.53 d), before either leaving the circulation or dying, as summarized graphically in Fig. 1 g.

**Table 1.** Derived variables for in vivo human monocyte kinetics

Subject	Proliferation		Delay		Lifespans					Pool sizes				Percentage transiting	
	**BM**		**BM to blood**		**marrow**	**CM**	**IM**	**NCM**		**CM**	**IM**	**NCM**		**CM→IM**	**IM→NCM**
	*per d*		*d*		*d*	*d*	*d*	*d*		*%*	*%*	*%*		*%*	*%*
Subject 1	0.48		1.53		1.04	1.37	4.29	6.44		80	8	12		3.2	100
Subject 2	0.28		1.70		1.77	0.5	5.26	7.52		83	7	10		0.8	100
Subject 3	0.26		1.61		1.9	0.62	3.55	8.28		90	3	7		0.6	100
Subject 4	0.64		1.70		0.78	1.54	4.11	N/R		96	2	2		0.8	N/R
**Mean**	**0.42**		**1.64**		**1.37**	**1.01**	**4.30**	**7.41**		**87**	**5**	**8**		**1.4**	**100**
SEM	0.09		0.04		0.27	0.26	0.36	0.53		3.59	1.47	2.17		0.62	0

Proliferation rate, lifespans, delays, and percentage of monocyte transitioning between subpopulations by subject for the model in which Δ_3_ = 0. Pool sizes were determined by flow cytometric analysis and were an input variable in the model. CMs, classical monocytes; IMs, intermediate monocytes; NCMs, nonclassical monocytes; N/R, data fit not resolved due to low labeling rates in nonclassical monocytes in subject 4.

Other studies have found evidence for a delay between intermediate monocytes and nonclassical monocytes (Tak, T., et al. 2016 British Society of Immunology/Dutch Society for Immunology Congress. Poster P207). We therefore investigated the consequences of including such a delay (Δ_3_) in our model. The goodness of the fits (ssr) were very similar with or without a delay. However, in three of four subjects, the model without Δ_3_ outperformed the model with Δ_3_ in terms of the corrected Akaike information criterion (Table S1). The estimates of monocyte lifetime were very similar for models with or without Δ_3_ (Table S2).

Our data are consistent with earlier murine studies, which provided evidence that the lifespan of each monocyte subpopulation varies; classical Ly6C^hi^ monocytes have shorter circulating half-lives (20 h) than nonclassical Ly6C^lo^ monocytes (2.2 d; Yona et al., 2013). The difference in circulating half-life between monocyte subsets is likely to correlate with their functional attributes. Classical monocytes replenish the large resident monocyte-derived population of the gut (Bain et al., 2014) and skin (Tamoutounour et al., 2013) and are poised to migrate to sites of inflammation, where they display a pro- or antiinflammatory phenotype depending on microenvironmental cues (Mildner et al., 2013b). More recently, these cells have been shown to enter tissues under steady state and transport antigen to lymph nodes without differentiating (Jakubzick et al., 2013). Less is known regarding the fate of nonclassical monocytes, but it is well documented that mouse and human nonclassical monocytes patrol the endothelium (Auffray et al., 2007; Cros et al., 2010) and represent a more terminally differentiated blood resident monocyte–derived cell.

### Human endotoxemia provokes the early release of bone marrow monocytes

We next investigated the response of monocytes to major systemic inflammation using the human experimental endotoxemia model (Fig. 2 a; Fullerton et al., 2016). A single i.v. injection of endotoxin induced a profound acute monocytopenia, following which, population numbers in blood recovered rapidly (Fig. 2, b and c). Corresponding in vitro studies have reported functional differences in the response to LPS between monocyte subpopulations (Cros et al., 2010).

Volunteers challenged with 2 ng/kg endotoxin (Fig. 2 a) experienced a complete loss of circulating Lin^−^ HLA-DR^+^ cells within the first 2 h after receiving endotoxin (Fig. 2 b). Strikingly, repopulation of the blood monocyte pool began very rapidly. Classical monocytes were the first subset to repopulate the circulation and appeared as early as 4 h after endotoxin; intermediate and nonclassical monocytes remained absent from the circulation until 24 h (Fig. 2 b). By day 7, monocyte numbers had returned to steady-state values (Fig. 2 c).

These data are consistent with previous studies in rodents, in which there is an expansion in circulating classical Ly6C^hi^ monocytes following both peripheral and systemic inflammation (Shi et al., 2011; Griseri et al., 2012; Heidt et al., 2014). Interestingly, the recovery surge of monocyte subsets following systemic inflammation recapitulates the order in which deuterium labeling appeared in monocyte subsets in healthy homeostasis (Fig. 1 d).

We set out to address whether classical monocytes marginate and then return to the circulation or whether their reappearance is due to an early “emergency” release from the bone marrow monocyte pool. To address this question, volunteers were pulsed with deuterium-labeled glucose 20 h before endotoxin challenge. We deliberately chose this time point preendotoxin, as we knew from the healthy labeling data that at this time point after labeling, no circulating monocytes would normally be labeled (Fig. 1 d), whereas cells in the postmitotic phase within the bone marrow pool could be expected to be highly labeled. Hence, unlabeled cells reappearing from margination could be readily distinguished from highly labeled cells released early from bone marrow.

We observed very high levels of deuterium labeling in classical monocytes at 8 h following endotoxin challenge (Fig. 2 d), demonstrating that these cells must have been recently released from the bone marrow. Although it cannot be confirmed that all classical monocytes were released from the bone marrow, due to the limitations of human experimentation, the fraction labeled were very similar to those seen 72 h after labeling in healthy homeostasis and are consistent with the proposal that most, if not all, circulating monocytes in the early recovery phase are bone marrow derived, rather than monocytes returning from a marginated pool. Certainly, it is clear that the transition time from bone marrow to the circulation is reduced dramatically in comparison to steady state as a result of the emergency release of classical monocytes.

### Classical human monocytes have the potential to give rise to intermediate and nonclassical monocytes

**Figure 3.**
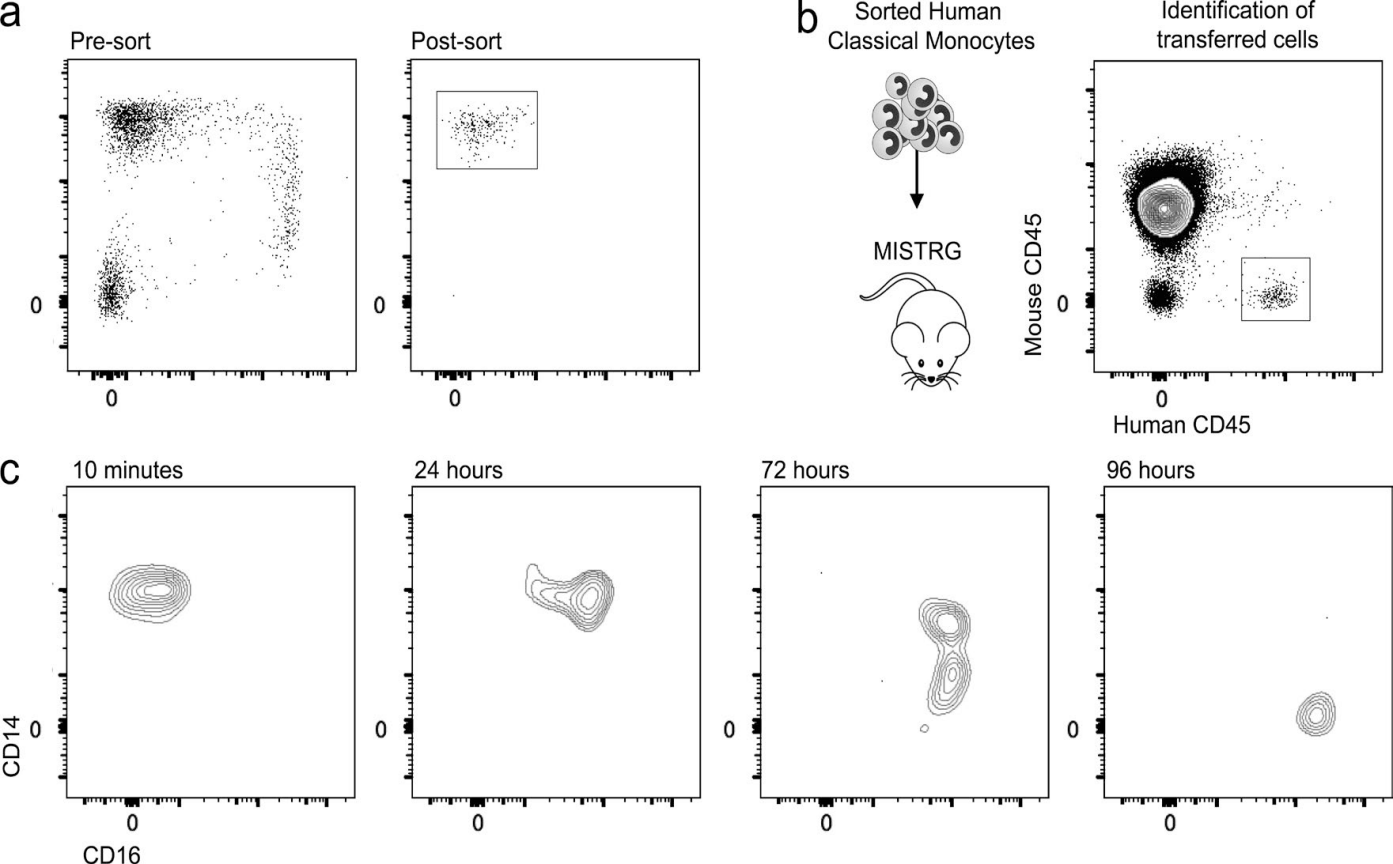
**Development of intermediate and nonclassical human monocytes from classical monocytes**. (a) Classical human monocyte LIN^−^ HLA-DR^+^ CD14^+^ CD16^−^ cells were sorted from healthy blood by FACS. (b) 1.5 × 10^6^ sorted classical monocytes were grafted i.v. into the humanized MISTRG mouse. Grafted cells could be readily identified by expression of the human isoform of CD45 compared with recipient leukocytes expressing mouse CD45. (c) Flow cytometry analysis identified human CD45^+^ circulating monocytes from MISTRG recipients following adoptive transfer of human CD14^+^CD16^−^ classical monocytes at 10 min and 24, 72, and 96 h after infusion. Results are representative of three analyzed mice per time point.

Given the sequential maturation of monocyte subsets during healthy homeostasis and, reappearance of monocytes following endotoxin challenge, we investigated the developmental relationship between human monocytes subsets in a humanized animal model. To this end, we analyzed the fate of classical human monocytes isolated from healthy volunteers and grafted into MISTRG mice (Fig. 3). The MISTRG mouse is a novel humanized mouse containing human versions of five genes encoding the cytokines thrombopoietin, IL-3, CSF2 (GM-CSF), SIRPα, and CSF1 (M-CSF) that help maintain human mononuclear phagocyte development (Rongvaux et al., 2014; Deng et al., 2015). Recipient mice were sacrificed at various time points following transfer, and peripheral blood was subjected to flow cytometry analysis. 10 min after transfer engraftment, (human) CD45^+^ cells detected in recipient blood displayed a classical monocyte phenotype; by 24 h, the grafted cells had transitioned to intermediate monocytes, and by 96 h, all grafted cells were nonclassical monocytes (Fig. 3 c). Collectively, this establishes for the first time that human classical monocytes have the potential to become intermediate monocytes before finally differentiating into nonclassical monocytes in vivo. These studies are reminiscent of previous rodent experiments, where classical Ly6C^hi^ monocytes were shown to convert into nonclassical cells over time (Varol et al., 2007; Yona et al., 2013; Gamrekelashvili et al., 2016). Although the conversion times differed from those seen in the in vivo deuterium-labeling studies, this is most likely due to grafted cells already being mature classical monocytes. A recent murine study has demonstrated Notch2 signaling is required for classical Ly6C^+^ monocytes to convert to nonclassical monocytes (Gamrekelashvili et al., 2016). Due to the challenging nature of ex vivo monocyte culture, this has not been demonstrated in human cells, but hopefully, future advances in cell culture will enable us to fully comprehend the mechanisms involved in human monocyte conversion.

Collectively, these data suggest that monocyte precursors first differentiate into classical monocytes that are retained in marrow for a postmitotic maturation phase of ∼38 h. As a result of this delay, a reserve population of newly generated classical monocytes is retained in bone marrow. Following acute systemic inflammation, this reserve population is rapidly released to replace lost circulating cells. Once in the circulation, both in vivo modeling and humanized animal experiments are most consistent with a model in which most classical monocytes leave the circulation after a circulating lifespan of ∼1 d. A small proportion of classical monocytes further mature into intermediate monocytes in the circulation; most of these cells finally convert to nonclassical monocytes before leaving the circulation. Clearly, this is a very tightly controlled process, with remarkably consistent results between individuals. Establishing the regulatory mechanisms that control these processes will be the next step in exploring human monocyte biology regulation. Understanding the fundamental regulation of monocyte subset generation, differentiation, and function will dictate future therapeutic avenues, depleting them when they are detrimental and boosting them when they are beneficial.

## Materials and methods

### Subjects and ethics

Subjects were healthy volunteers (20 males and 5 females). All volunteers gave written informed consent, and all studies were conducted according to the principles of the declaration of Helsinki after approval by the relevant institutional review boards (for deuterium and steady-state experiments, NRES Committee West London [10/H0803/102] and University College London Research Ethics Committee [p8081/001], and for the endotoxemia study [5060/001]). Human bone marrow samples were obtained from hematopoietic stem cell donors or femoral heads following total hip replacement. Newcastle and North Tyneside Research Ethics Committee approved the bone marrow biopsy (REC 14/NE/113) and hip (REC 14/NE/1212) procedures.

### Flow cytometry and cell sorting

PBMCs were isolated by Ficoll-Paque Plus (GE Healthcare) by density centrifugation (1,000 *g,* low acceleration, no brake) and then resuspended in PBS containing 2% FCS and 2 mM EDTA. Isolated PBMCs were incubated with Human Trustain FcX (BioLegend) before labeling with the following antibodies obtained from BioLegend (unless otherwise stated): CD3 (HIT3a), CD11b (ICRF44), CD11c (B-ly6; BD), CD14 (M5E2), CD16 (3G8), CD19 (HIB19), CD20 (2H7), CD33 (WM53), CD36 (5–271), hCD45 (H130), mCD45 (30F11), CD56 (MEM-188), CD62L (DREG-56), CD64 (10.1), CD66b (G10F5), HLA-DR (G46-6; BD), CX_3_CR1 (2A9-1), CCR2 (KO36C2), and SLAN (MDC-8; Miltenyi Biotec). DAPI staining was performed in specified experiments following surface staining, and cells were fixed and permeabilized in Fixation Buffer and Intracellular Staining Permeabilization Wash Buffer (BioLegend) according to the manufacturer’s instructions before incubation with 0.05 ng/ml DAPI. For a positive control, the human monocyte cell line Mono Mac 6 was used (Ziegler-Heitbrock et al., 1988). For bone marrow isolation, cells from hip arthroplasty specimens and bone fragments were excavated from femoral heads. The cavity and fragments were washed with PBS and filtered through a 50-µm filter. Mononuclear cells were prepared from the resulting cell suspension or bone marrow aspirate from hematopoietic stem cells healthy donors by density centrifugation as described for PBMCs. Cells were stained for CD3 (SK7-Leu9; BD), CD19 (HIB19; BD), CD20 (L27; BD), CD7 (4H9; BD), CD14 (M5E2; BioLegend), CD16 (3G8; BD), HLA-DR (G46-6; BD), and DAPI (Sysmex) for dead cell exclusion. Flow cytometry was performed with LSR Fortessa X20 (BD) and cell sorting by FACS Aria II (BD), and data were analyzed offline with FlowJo (Tree Star) and Cytobank (Cytobank, Inc.).

### Deuterium labeling

Deuterium labeling followed a shortened version of published protocols (Macallan et al., 2009; Westera et al., 2013). Subjects received 20 g deuterium-labeled glucose (6,6-^2^H_2_-glucose; Cambridge Isotopes) as an oral solution in half-hourly aliquots over 3 h, following a priming dose equivalent to 1.8 h dosing at time 0. Blood glucose enrichment was monitored at baseline, during and after labeling. At selected time points after labeling, mononuclear phagocytes subsets were stained and sorted by FACS Aria II (BD), DNA extracted, and deuterium enrichment measured by gas chromatography mass spectrometry, as previously described (Busch et al., 2007; Macallan et al., 2009).

### Modeling of data

A schematic of the model is shown in Fig. 1 e. We denote N as the number of monocytes in the bone marrow, B_1_ the number of classical monocytes in the blood, B_2_ the number of intermediate monocytes in the blood, and B_3_ the number of nonclassical monocytes in the blood. The dynamics between these four compartments can then described by the following equations:

$$\frac{dN}{dt}=pN-r_{1}N$$

$$\frac{dB_{1}}{dt}=r_{1}N\left(t-\;\Delta_{1}\right)-r_{2}B_{1}$$

$$\frac{dB_{2}}{dt}=\alpha_{2}r_{2}B_{1}-r_{3}B_{2}$$

$$\frac{dB_{3}}{dt}=\alpha_{3}r_{3}B_{2}\left(t-\;\Delta_{3}\right)-r_{4}B_{3}$$

We assume that all the compartments are in steady state. The relative sizes of B_1_, B_2_, and B_3_ were taken from flow cytometry data for each individual (Table 1). From these equations, we derive the dynamics of the fraction of labeled cells in each compartment: F_N_ for the bone marrow and F_x_ for blood compartments B_x_:

$$\frac{dF_{N}}{dt}=pU\left(t\right)b-r_{1}F_{N}$$

$$\frac{dF_{1}}{dt}=r_{1}\frac{N}{B_{1}}F_{N}\left(t-\;\Delta_{1}\right)-r_{2}F_{1}$$

$$\frac{dF_{2}}{dt}=\alpha_{2}r_{2}\frac{B_{1}}{B_{2}}F_{1}-r_{3}F_{2}$$

$$\frac{dF_{3}}{dt}=\;\alpha_{3}r_{3}\frac{B_{2}}{B_{3}}F_{2}\left(t-\;\Delta_{3}\right)-r_{4}F_{3}$$

Here, U(t) is the precursor enrichment (plasma glucose) at time t, described empirically as a plateau function with exponential decay.

We used the R packages modFit and dede to fit the model to the observed values of deuterium enrichment (F_x_ in the equations above). The fitting algorithm sought to minimize the sum of squared residuals between the modeled curves and observed values. This sum of squared residuals was translated into an Akaike information criterion (corrected for small sample sizes), allowing us to compare the models with and without Δ_3_ (because a model with more parameters will trivially result in an equal or better fit but comes with a risk of overfitting).

#### Intravenous administration of endotoxin

2 ng/kg endotoxin (*Escherichia coli* 0:113; National Institutes of Health Clinical Center) was administered i.v. to 10 healthy male volunteers as described previously (Fullerton et al., 2016). At selected time points, blood samples were taken and analyzed by flow cytometry. Three subjects received deuterium-labeled glucose 20 h before endotoxin administration and monocyte labeling kinetics analyzed as above (Fig. 2 a).

#### Mice

10-wk-old MISTRG mice (Rongvaux et al., 2014; Deng et al., 2015) were used for adoptive transfer experiments. Mice were maintained under specific pathogen–free conditions and handled under protocols approved by the Yale Institutional Animal Care and Use Committee.

#### Mouse adoptive transfer

Blood was collected from healthy volunteers and mononuclear phagocytes enriched using RosetteSep Human Monocyte Enrichment Cocktail (STEMCELL Technologies) following the manufacturer’s instructions. Enriched cells were labeled with CD3, CD19, CD20, CD56, CD66b, HLA-DR, CD14, and CD16 antibodies before sorting classical monocytes by FACS AriaII (BD). Sorted classical monocytes were adoptively transferred intravenously into MISTRG mice (Rongvaux et al., 2014; Deng et al., 2015). Peripheral blood was collected by cardiac puncture under terminal anesthesia; erythrocytes were lysed by ACK (Lonza). The leukocyte fraction was stained and analyzed by flow cytometry. The fate of the classical monocytes were analyzed at selected time points after transfer identified as human mCD45^−^hCD45^+^HLA-DR^+^CD33^+^Lin^−^ by flow cytometry.

### Online supplemental material

Fig. S1 quantifies human blood monocyte subset membrane marker expression and cell cycle analysis. Fig. S2 shows modeling curves generated with and without a delay between intermediate and nonclassical monocytes. Table S1 shows model data fit with and without a delay. Table S2 shows lifespans, proliferation rates, and delays for the model with a delay.

## Supplementary Material

Supplemental Materials (PDF)
