# Supplementary material for: The fate and lifespan of human monocyte subsets in steady state and systemic inflammation
Source: J Exp Med. 2017 Jul 3;214(7):1913–23. doi: 10.1084/jem.20170355 (PMC5502436; doi:10.1084/jem.20170355)
Supplement: Supplemental Materials (PDF) [file JEM_20170355_sm.pdf]

The Journal of Experimental Medicine

SUPPLEMENTAL MATERIAL

Patel et al., https://doi.org/10.1084/jem.20170355

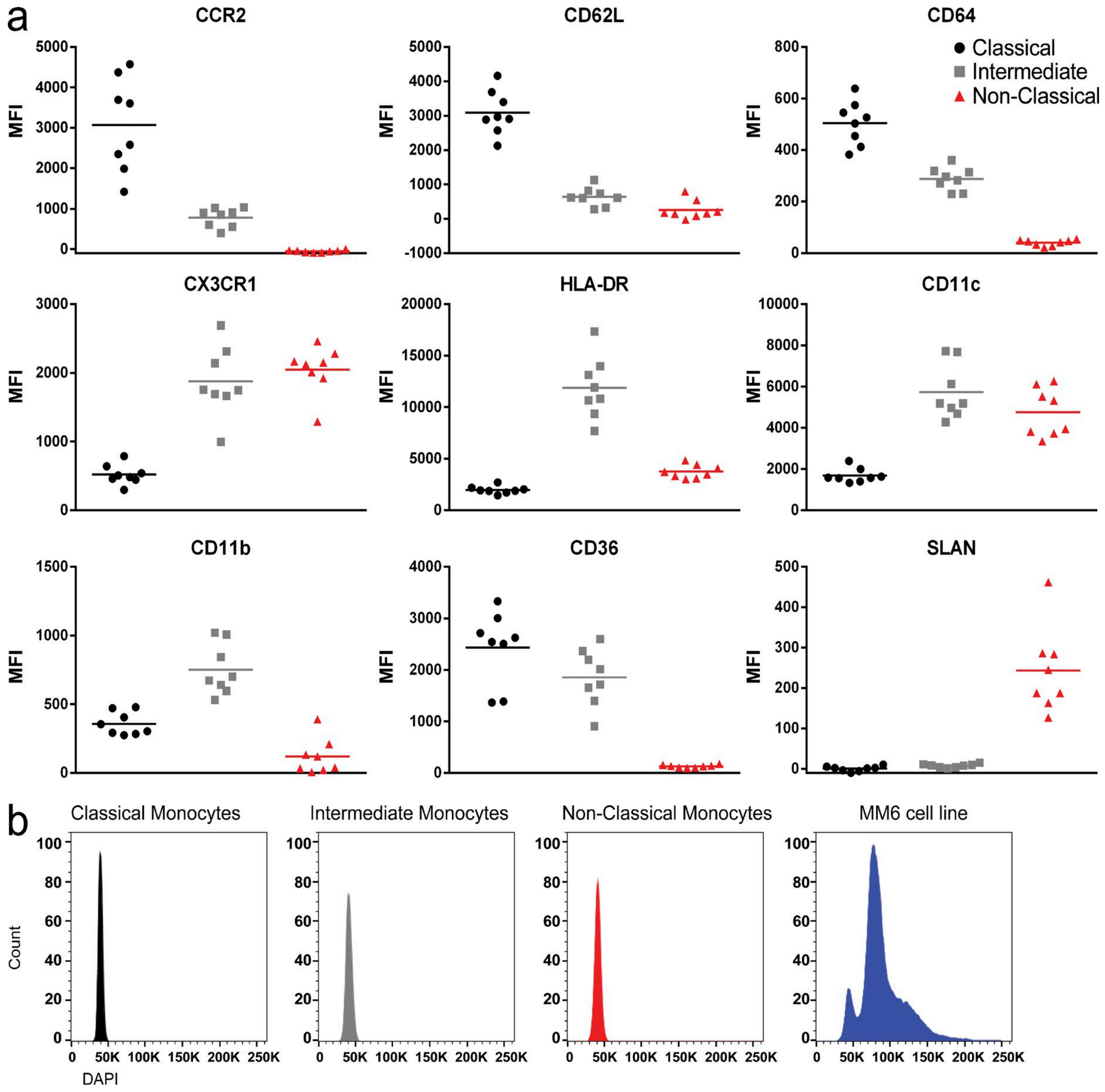

Figure S1. **Heterogeneity of membrane markers within monocyte subsets.** (a) Polychromatic flow cytometry analysis of circulating monocyte subsets membrane expression of CCR2, CD62L, CD64, CX_3CR1, HLA-DR, CD11c, CD11b, CD36, and SLAN from eight healthy volunteers. Bars represent mean MFI (mean fluorescence intensity). (b) Cell cycle analysis of circulating blood monocytes. DAPI was used to quantify DNA levels to determine whether circulating monocytes are in various phases of the cell cycle. The Mono Mac 6 (MM6) monocyte cell line was used as a positive control to show various stages of the cell cycle.

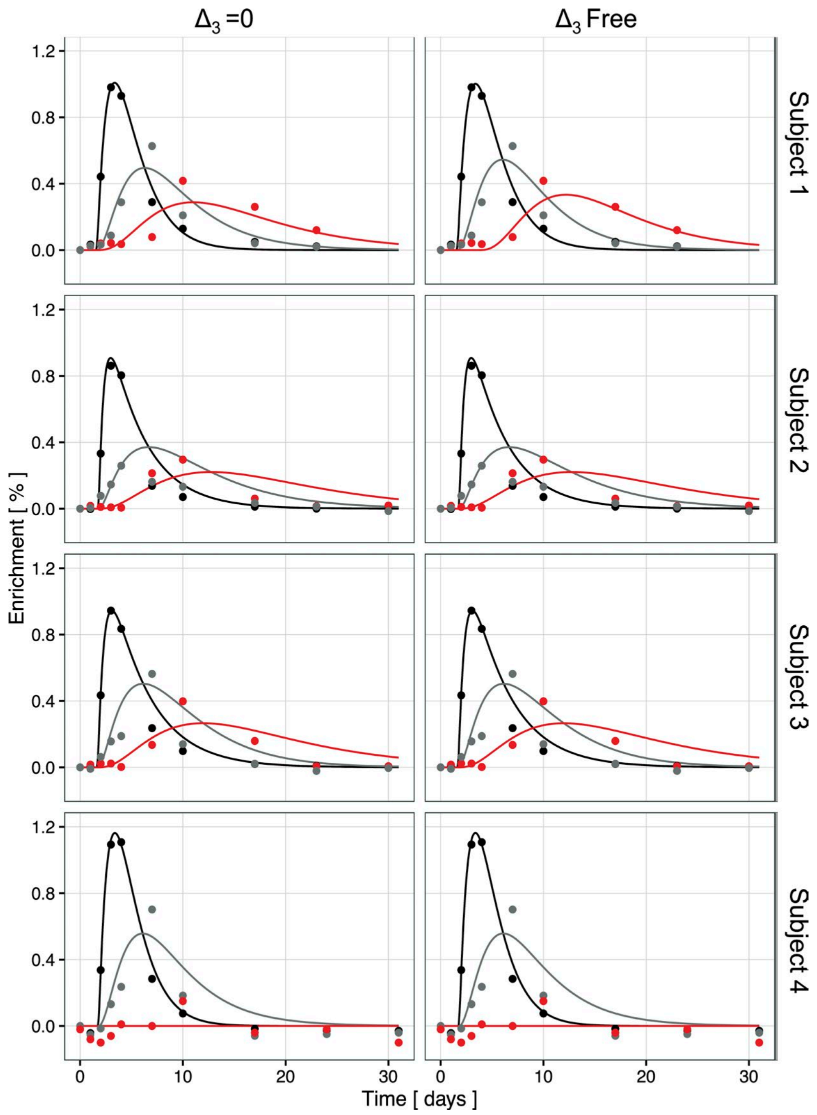

Figure S2. **Curve fits to experimental data from modeling with and without a delay between intermediate and nonclassical monocytes.** Dots represent experimental data expressed as percentage DNA enrichment with deuterium in the three sorted blood monocyte subsets. Lines represent the best model fit to the data classical monocytes (black lines), intermediate monocytes (gray lines), and nonclassical monocytes (red lines). Each row represents an individual subject. Columns represent alternative models for Δ_3 constrained to zero (left) or as a free parameter (right).

Table S1. **Comparison of model data fit with and without a delay between intermediate and nonclassical monocytes**

Subject	$\Delta_3 = 0$	Δ_3 free
**Subject 1**		
ssr	0.11	0.09
AICc	−137	−137.4
**Subject 2**		
ssr	0.185	0.185
AICc	−140.2	−137
**Subject 3**		
ssr	0.19	0.19
AICc	−139.3	−136.2
**Subject 4**		
ssr	0.18	0.18
AICc	−140.9	−137.7

Sum of squared residuals (ssr) and Akaike information criterion corrected (AICc; corrected for small sample sizes) for model fitted to experimental data as shown in Fig. 1 c, with Δ_3 constrained to zero or allowed to be a free parameter. As a rule of thumb, one model outperforms another if the AICc is at least three units lower (more negative) than the other.

Table S2. **Lifespans, proliferation rates, and delays for the best-fit models per subject allowing Δ_3 to be a free parameter**

Subject	Proliferation	Delay	Lifespans				Pool sizes			Δ_3
	**BM**	**BM to blood**	**marrow**	**CM**	**IM**	**NCM**	**CM**	**IM**	**NCM**	
	per d	*d*	*d*	*d*	*d*	*d*	%	%	%	*d*
Subject 1	0.50	1.53	1.01	1.44	3.55	5.33	80	8	12	2.1
Subject 2	0.28	1.70	1.77	0.35	5.25	7.51	83	7	10	<0.001
Subject 3	0.26	1.61	1.9	0.62	3.55	8.29	90	3	7	<0.001
Subject 4	0.64	1.70	0.78	1.54	4.11	N/R	96	2	2	N/R
Mean	0.42	1.64	1.37	0.99	4.11	7.04	87	5	8	-
SEM	0.09	0.04	0.28	0.30	0.40	0.89	3.59	1.47	2.17	-

Proliferation rate, lifespans, delays, and percentage of monocytes transitioning between subpopulations by subject for when Δ_3 is a free parameter. Pool sizes were determined by flow cytometric analysis and were an input variable in the model. Note that for subject 4, we do not find a reliable estimate for Δ_3, because there is little enrichment in the NCM compartment. Plots of the best fits are shown in Fig. S2. CMs, classical monocytes; IMs, intermediate monocytes; NCMs, nonclassical monocytes; N/R, not resolved.
